# Supplementary material for: A 3.4-Å cryo-electron microscopy structure of the human coronavirus spike trimer computationally derived from vitrified NL63 virus particles
Source: QRB Discov. 2020 Nov 17;1:e11. doi: 10.1017/qrd.2020.16 (PMC7737156; doi:10.1017/qrd.2020.16)
Supplement: Supplementary file 1 [file S2633289220000162sup001.pdf]

Supplementary Information

A 3.4-Å cryo-EM structure of the human coronavirus spike trimer computationally derived from vitrified NL63 virus particles

Kaiming Zhang[1], Shanshan Li[1], Grigore Pintilie[1], David Chmielewski[2], Michael F. Schmid[4], Graham Simmons[3], Jing Jin[3] and Wah Chiu[1,4,*]

[1]Department of Bioengineering and James H. Clark Center, Stanford University, Stanford, CA 94305, USA

[2]Graduate Program in Biophysics, Stanford University, Stanford, CA 94305, USA

[3]Vitalant Research Institute, San Francisco, CA 94030, USA

[4]Division of CryoEM and Bioimaging, SSRL, SLAC National Accelerator Laboratory, Menlo Park, CA 94025, USA

[*]Correspondence: wahc@stanford.edu

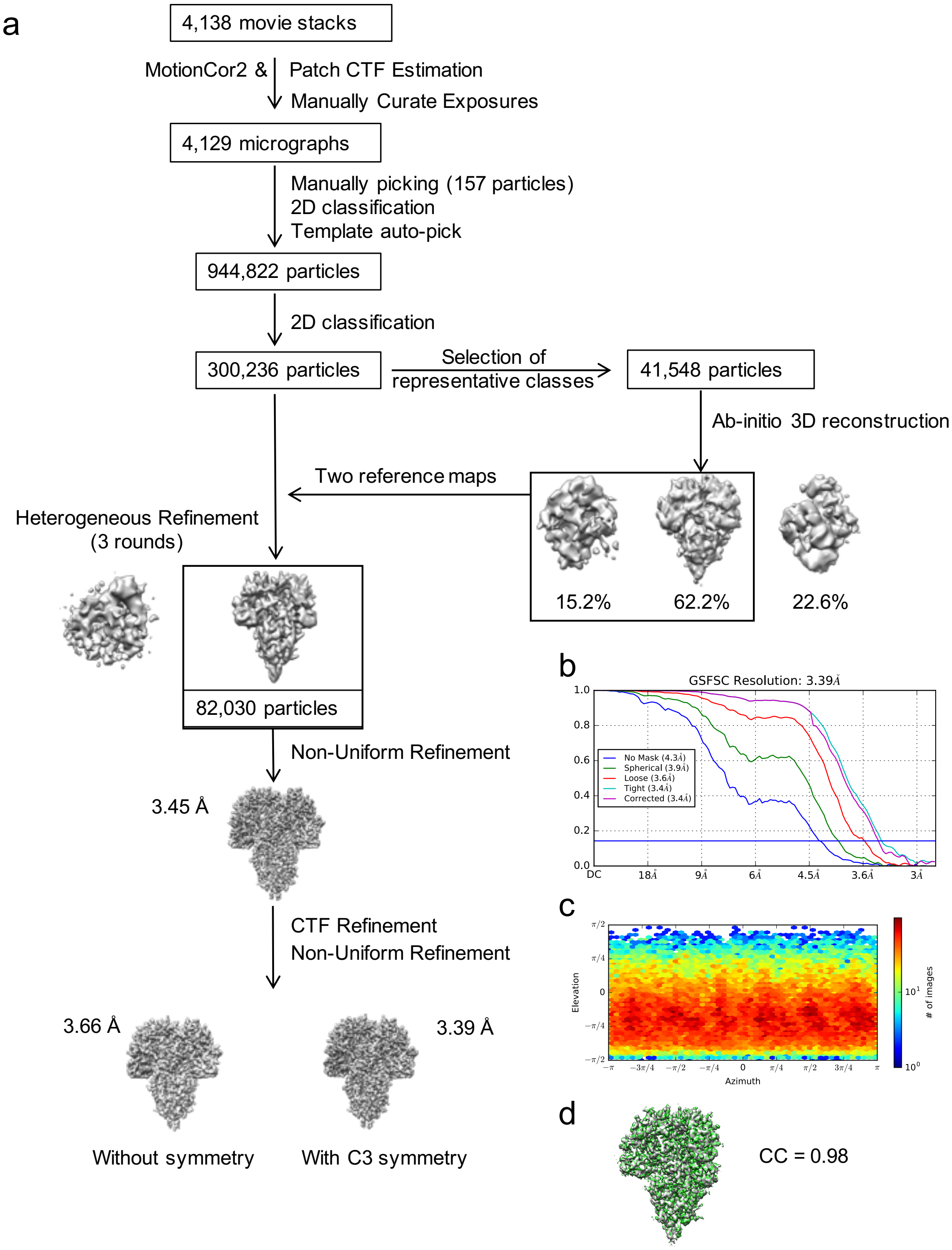

Fig. S1. Single-particle cryo-EM data processing of HCoV-NL63 coronavirus spike glycoprotein. (*a*). Workflow of the data processing. (*b*) Gold standard FSC plot for the final 3D reconstruction. (*c*) Euler angle distribution of the particle images of computationally extracted spikes, calculated in cryoSPARC. (*d*) Cross-correlation coefficient between the 3D reconstructions with (green) and without (grey) C3 symmetry applied.

CoV2 Domain A’ to NL63 Domain 0

```
Aligned length=159, RMSD=3.48Å, Identity (#identical/#aligned)=7.5%
TM-score= 0.58993 (if normalized by length of Chain_1, i.e., LN=203, d0=5.30)
TM-score= 0.59225 (if normalized by length of Chain_2, i.e., LN=202, d0=5.29)

(":" denotes residue pairs of d <  4.0 Angstrom, "." denotes other aligned residues)

CoV2_A  AYTNSFTRGVYYPDKVFRSSVLHSTQDLFLPFFSNVTWFHAIHDNPVLPFNDGVYFASTEKSNIIRGWIFGTTLD
               :........:::::::::::::  . ...::.  :::::::  :::::..::::: :::::::..:
NL63_0  -------ANLSMLQLGVPDNSSTIVTGLLPT--H-WFCANQ--STSVYSA--NGFFYIDVGNHRS-AFALHTGYY

CoV2_A  SK-TQSLLIVNNAT---NVVIKVCEFQFCNDPFLGV---NCTFEYVSFK---NLREFVFKNIDGYFKIYSKHTPI
        .: ::::::::...   .:::::::::..          ::::::.     ...::::::::::::::.
NL63_0  DANQYYIYVTNEIGLNASVTLKICKFSRS-------SSFDCIVNLLF--TEQLGAPLGITISGETVRLHL-----

CoV2_A  NLVRDLPQGFSALEPLVDLPIG-I-NITRFQTLLALH--A-AYYVGYLQPRTFLLKYNENGTITDAVD-------
                    ....:::::: : ::::::::...    : ...::::::::::::::: :::::::
NL63_0  ------------YNVTRTFYVPAAYKLTKLSVKCY--FNYSCVFSVVNATVTVNVTTHNG-RVVNYTVCDDCNGY

CoV2_A  --------------------

NL63_0  TDNIFSVQQDGRIPNGFPFNN
```

CoV2 Domain A’ to NL63 Domain A

```
Aligned length=157, RMSD=3.72Å, Identity (#identical/#aligned)=10.2%
TM-score= 0.57667 (if normalized by length of Chain_1, i.e., LN=203, d0=5.30)
TM-score= 0.56989 (if normalized by length of Chain_2, i.e., LN=206, d0=5.34)

(":" denotes residue pairs of d <  4.0 Angstrom, "." denotes other aligned residues)

CoV2_A  AYTNSFTRGVYYPDKVFRSSVLHSTQDLFLPFFSNVTWFHAIH-DNPVLPF-----------------NDGVYFAS
               .:.:.:.:.....::::::::::.:. .::.:::..  .::::::                 .:::::::
NL63_A  -------NWFLLTNGSTLVDGVSRLYQPLRLT-CLWPVPGLK-SSTGFVYFNATGSDVNCNGYQHNSVVDVMRYNL

CoV2_A  TEK----SN-IIRGWIFGTTLDSKTQSLLIVN---------N-A-----T-NVVIKVCEFQFCNDPFLGV-NCTFE
        ::.    :: :..:::::.   .:::::::.          : .     . ::::::::..             ....:
NL63_A  NFSANSLDNLKSGVIVFKTL---QYDVLFYCSNSSSGVLDTTIPFGPSSQPYYCFINSTIN---------TTHVST

CoV2_A  YV-SFK---NLREFVFKNIDGYFKIYSKHTPINLVRDLPQGFSALEPLVDLPIGINITRFQTLLALH--AAY-YVG
        :. .     ..::::::.. . :::::                  :: :::::: ::::::::::..  ::: :::
NL63_A  FVGI--LPPTVREIVVART-G-QFYIN------------------GF-KYFDLG-FIEAVNFNVTTASATDFWTVA

CoV2_A  YLQPRTFLLKYNENGTITDAVD--
        ::::::::::::. ....:::::
NL63_A  FATFVDVLVNVS-ATNIQNLLYCD
```

NL63 Domain A to NL63 Domain 0

```
Aligned length=160, RMSD=3.88Å, Identity(#identical/#aligned)=8.1%
TM-score= 0.54596 (if normalized by length of Chain_1, i.e., LN=206, d0=5.34)
TM-score= 0.55398 (if normalized by length of Chain_2, i.e., LN=202, d0=5.29)

(":" denotes residue pairs of d <  4.0 Angstrom, "." denotes other aligned residues)

CoV2_B  NWFLLTNGSTLVDGVSRLYQ-PLRLTCLWPVPGLKSSTGFVYFNATGSDVNCNGYQHNSVVDVMRYNLNFSANSLD
        .::.... ....:::.:::: ....  :..:::.:  ::::::                  ..::: :.:
NL63_B  ANLSMLQ-LGVPDNSSTIVTGLLPT--HWFCANQS--TSVYSA-----------------NGFFY-IDV------

CoV2_B  NLKSGVIVFKT---L--QYDVLFYCSNSSSGVLDTTIPFGPSSQPYYCFINSTIN---TTHVSTFVGILPPTVREI
        ::::::::::.   .  :::::::            .::   .:..:.:::.::..   .:.:.:::.::::::::
NL63_B  GNHRSAFALHTGYYDANQYYIYVT-----------NEI---GLNASVTLKICKFSRSSSFDCIVNLLFTEQLGAPL

CoV2_B  VVART---GQFYINGFKYFD-L--GF-IEAVNFNVTTASATDFWTVAFATFVDVLVNVSATNIQNLL-Y-CD----
        ::::.:   ::::::.::..: .  :: :::::::. . .:.::..:.:.:...:::.:::::.. . .:
NL63_B  GITISGETVRLHLYNVTRTFYVPAAYKLTKLSVKC-Y-FNYSCVFSVVNATVTVNVTTHNGRVVNYTVCDDCNGYT

CoV2_B  --------------------

NL63_B  DNIFSVQQDGRIPNGFPFNN
```

CoV2 Domain B’ to NL63 Domain B

```
Aligned length=95, RMSD=4.13Å, Identity (#identical/#aligned)=9.5%
TM-score= 0.38757 (if normalized by length of Chain_1, i.e., LN=168, d0=4.83)
TM-score= 0.44403 (if normalized by length of Chain_2, i.e., LN=140, d0=4.40)

(":" denotes residue pairs of d <  4.0 Angstrom, "." denotes other aligned residues)

CoV2_B  -ITNLCPFGEVFNAT---------------RFASVYA---WNRKRISNCVADYSVLYNS---------------AS
         :::::: :  ::..                ::.        ..::::::.:::. ..                ..
NL63_B  YYQHTDI-N--FTATASFGGSCYVCKPHQVNIS----LNGNTSVCVRTSHFSI-RY---IYNRVKSGSPGDSSWHI

CoV2_B  FSTFKCYGVSPTKL-NDLCFTNVYADSFVIRGDEVRQIAPGQTGKIADYNYKLPDDFTGCVIAWNSNNLDSKGNYN
        :.:::.:::....: :::::::::::::                       ...:  . ..:.:::..
NL63_B  YLKSGTCPFSFSKLNNFQKFKTICFSTV----------------------EVPG--S-CNFPLEATW---------

CoV2_B  YLYRKPFERDIYFPLQSYGFQPT---NVGYQPYRVVVLSFELLHAPATVCGPK-------K
                                  ..:  .:::::::::: : .: ::::.       .
NL63_B  ----------------------HYTSYT--IVGALYVTWSE-G-NS-ITGVPYPVSGIRE
```

CoV2 Domain C’ to NL63 Domain C

```
Aligned length=59, RMSD=1.96Å, Identity (#identical/#aligned)=25.4%
TM-score= 0.71304 (if normalized by length of Chain_1, i.e., LN=61, d0=2.64)
TM-score= 0.72167 (if normalized by length of Chain_2, i.e., LN=60, d0=2.61)

(":" denotes residue pairs of d <  4.0 Angstrom, "." denotes other aligned
residues)

CoV2_C  STNLVKNKCVNFNFNGLTGTGVLTESNKKFLPFQQFGRDIADTTDAVRDPQTL-EILDITPC
        :::::::::::::::::::::::::::::: :::::::.:::::::. .: ::::::::
NL63_C  FSNLVLNNCTKYNIYDYVGTGIIRSSNQSLA-GGITYVSNSGNLLGFKNV-STGNIFIVTPC
```

CoV2 Domain S2’ to NL63 Domain S2

```
Aligned length=416, RMSD=3.10Å, Identity (#identical/#aligned)=37.0%
TM-score= 0.87726 (if normalized by length of Chain_1, i.e., LN=422, d0=7.39)
TM-score= 0.80337 (if normalized by length of Chain_2, i.e., LN=464, d0=7.70)

(":" denotes residue pairs of d <  4.0 Angstrom, "." denotes other aligned residues)

CoV2_S2  --GAENSVA-YS-N-NSIAIPTNFTISVTTEILPVSMTKTSVDCTMYICGDSTECSNLLLQYGSFCTQLNRALTG
           ::..... .. . :.::::::::::::::::::::::::::::::::::::::::::::::::::::::::
NL63_S2  RPRNSSDNGISAIITANLSIPSNWTTSVQVEYLQITSTPIVVDCATYVCNGNPRCKNLLKQYTSACKTIEDALRL

CoV2_S2  IAVEQDKNTQEVFAQVKQIYKTPPIKDFGGFNFSQILPDPSKP----SKRSFIEDLLFNKVT------------
         :::::::.::..::.....:::...:.:.:::::::::::....    .::::::::::::::
NL63_S2  SAHLETNDVSSMLTFDSNAFSLANVTSFGDYNLSSVLPQRNIRSSRIAGRSALEDLLFSKVVDVDYKSCTKGLSI

CoV2_S2  ------K-FNGLTVLPPLLTDEMIAQYTSALLAGTITSGWTFGAGAALQIPFAMQMAYRFNGIGVTQNVLYENQ
               : :::::::::::::::::::::::::::: ::. ..  ...::::::::::::::::.:.:..::::
NL63_S2  ADLACAQYYNGIMVLPGVADAERMAMYTGSLIGGM-VLG-GL--TSAAAIPFSLALQARLNYVALQTDVLQENQ

CoV2_S2  KLIANQFNSAIGKIQDSLSS------TASALGKLQDVVNQNAQALNTLVKQLSSNFGAISSVLNDILSRLDPPE
         ::::::::::::::::::::      ...:::::::::::::::::::::::::::::::::::::::::::
NL63_S2  KILAASFNKAINNIVASFSSAEAIHTVTIALNKIQDVVNQQGSALNHLTSQLRHNFQAISNSIQAIYDRLDSIQ

CoV2_S2  AEVQIDRLITGRLQSLQTYVTQQLIRAAEIRASANLAATKMSECVLGQSKRVDFCGKGYHLMSFPQSAPHGVVF
         ::::::::::::::::::::::::::::::::::::::::::::::::::::::::::::::::::::::::::
NL63_S2  ADQQVDRLITGRLAALNAFVSQVLNKYTEVRGSRRLAQQKINECVKSQSNRYGFCGNGTHIFSIVNSAPDGLLF

CoV2_S2  LHVTYVPAQEKNFTTAPAICHD-GKAHFPRE--GVFVSNGTHWFVTQRNFYEPQIITTDNTFVSGNCDVVIGIV
         :::::::::::::.::::::::: :::::::..  ::::.........::::::::::...:. :::::::: :.:::
NL63_S2  LHTVLLPTDYKNVKAWSGICVDGIYGYVLRQPNLVLYSDNGVFRVTSRVMFQPRLPVLS-DFVQIYNC-NVTFV

CoV2_S2  NN----T--VYDPLQPE--L--DS
         :.    .  ::::....  .  ..
NL63_S2  NISRVELHTVIPDYVDVNKTLQEF
```

CoV2 Domain D’ to NL63 Domain D

```
Aligned length=63, RMSD=3.10Å, Identity (#identical/#aligned)=6.3%
TM-score= 0.53671 (if normalized by length of Chain_1, i.e., LN=77, d0=3.11)
TM-score= 0.58449 (if normalized by length of Chain_2, i.e., LN=68, d0=2.86)

(":" denotes residue pairs of d <  4.0 Angstrom, "." denotes other aligned residues)

CoV2_D  SFGGVSVITPGTNTSNQVAVLYQDVNCTE-V----NVFQTRAGCLIGAEHVNNSYECDIPIGAGICASY
        ...::::::::   :::::::  :.:.  .    :::::.::::.:.:...: .::::: ::::::
NL63_D  CNQPDQVAVYQQ---SIIGAMT--AVNE-SRYGLQNLLQLPNFYYVSNGGNNCTT-AVMTYS-NFGICA

CoV2_D  QTSQSIIAYTMSL
            :::::::
NL63_D  ----DGSLIPV--
```

Fig. S2. Structure-based alignment of domains of spike protein from HCoV-NL63 and SARS-CoV-2. The method of TM-align was used, yielding good structural alignments illustrated in Fig. 5. Aligned sequences along with RMSD, percent identity, and TM-score are shown for all domain pairs.

Table S1. Cryo-EM data collection, refinement, and validation statistics.

	HCoV-NL63 S protein (EMDB-22889) (PDB ID: 7KIP)
**Data collection and processing**	
Magnification	64k
Voltage (kV)	300
Electron exposure ($e^{-}/Å^2$)	48
Defocus range (μm)	-0.4 to -3.6
Pixel size (Å)	1.4
Symmetry imposed	C3
Initial particle images (no.)	944,822
Final particle images (no.)	82,030
Map resolution (Å)	3.39
FSC threshold	0.143
Map resolution range (Å)	2.7-7.0
**Refinement**	
Initial model used (PDB code)	5SZS
Map sharpening *B* factor ($Å^2$)	-90
Model composition	
Non-hydrogen atoms	30,612
Protein residues	3,576
Ligands	222
B factors ($Å^2$)	
Protein	94.84
Ligand	141.21
R.M.S. deviations	
Bond lengths (Å)	0.009
Bond angles (°)	1.30
Validation	
MolProbity score	1.44
Clashscore	1.88
Poor rotamers (%)	0
Ramachandran plot	
Favored (%)	92.06
Allowed (%)	7.86
Disallowed (%)	0.08

Table S2. Comparison of the HCoV-NL63 S glycans in different studies.

Amino acid residue site	Sequon	MS identified (Walls et al)	Cryo-EM observed (Walls et al)	Cryo-EM observed (this study)
24	NLSM	ND	ND	24
35	NSST	35	35	35
52	NQST	52	52	52
98	NASV	98	98	98
111	NTTF	ND	ND	ND
119	NASS	ND	ND	ND
155	NVTR	155	155	155
178	NYSC	ND	ND	ND
187	NATV	187	187	187
193	NVTT	193	193	193
203	NYTV	ND	ND	203
240	NGST	240	240	240
276	NATG	276	276	276
301	NFSA	301	301	301
330	NSSS	ND	330	330
354	NSTI	ND	354	354
358	NTTH	358	358	358
403	NVTT	403	403	403
426	NVSA	ND	426	426
486	NFTA	486	486	486
506	NISL	506	506	506
512	NTSV	512	512	512
626	NCTK	626	626	626
645	NQSL	645	645	645
666	NVST	666	666	666
699	NESR	699	699	699
723	NCTT	ND	723	723
749	NSSD	ND	749	749
762	NLSI	ND	762	762
768	NWTT	768	768	768
844	NVTS	844	844	844
852	NLSS	852	852	852
1111	NGTH	1111	1111	1111
1196	NVTF	1196	1196	1196
1201	NISR	1201	1201	1201
1218	NKTL	1218	1218	1218
1242	NLTY	1242	ND	ND
1247	NLSS	1247	ND	ND
1277	NSTY	1277	ND	ND
**TOTAL # of sites**				
39		28	31	33
